# Early Palliative Care in Heart Transplant Evaluation

**DOI:** 10.7759/cureus.98804

**Published:** 2025-12-09

**Authors:** William S Stringer, Alissa A Ulanday, Christine M Bui, Rachel M Verhagen, Shelley J Choi

**Affiliations:** 1 Medicine, University of California Los Angeles David Geffen School of Medicine, Los Angeles, USA; 2 Nursing, University of California Los Angeles, Los Angeles, USA; 3 Palliative Care, University of California Los Angeles David Geffen School of Medicine, Los Angeles, USA; 4 Behavioral Health, University of California Los Angeles David Geffen School of Medicine, Los Angeles, USA

**Keywords:** claustrophobia, goals of care, orthotopic heart transplantation, palliative care, transplant candidacy, transplant evaluation

## Abstract

Palliative care (PC) is becoming increasingly integrated into the care of patients with advanced heart failure, although it is not yet widely incorporated into the heart transplant evaluation process. Patients undergoing evaluation for heart transplant carry a high burden of mental health morbidity, which can affect their ability to complete the evaluation and undergo successful transplantation. We describe the case of a 67-year-old male with ischemic cardiomyopathy on continuous inotropic support for decompensated heart failure, who initially declined to complete a transplant evaluation and expressed a desire for immediate hospital discharge. PC involvement clarified his prognosis without advanced heart failure therapies, and identified severe claustrophobia as the driver of his desire to end the transplant evaluation. With appropriate psychologic and psychiatric support, his claustrophobia became tolerable, and he underwent successful heart transplantation. With expertise in symptom management and effective communication, PC plays a crucial role as a bridge between medical and psychosocial teams, particularly when psychopathology is present. Appropriate PC involvement in the transplant evaluation process can identify and navigate barriers, and ensure goal-concordant, patient-centered care.

## Introduction

Palliative care (PC) is an increasingly well-established treatment paradigm for end-stage organ failure [1]. Alleviating suffering through complex symptom management and advocating for goal-concordant care makes PC well-suited to address the high burden of symptoms, morbidity, and mortality associated with end-stage organ disease [2-5]. PC in advanced heart failure is an emerging field, with randomized trials and large observational studies demonstrating a link between PC and improvement in the quality of life and healthcare utilization for these patients, but not mortality benefit [2,6]. The American College of Cardiology/American Heart Association/Heart Failure Society of America (ACC/AHA/HFSA) 2022 Heart Failure Guidelines advocate for early and routine involvement of PC for those with advanced heart failure, including those undergoing evaluation for advanced therapies [7]. In clinical practice, PC is not commonly involved in the evaluation for solid organ transplant (SOT), but it is often integrated into patient care when attempts at advanced therapies have been unsuccessful and a patient is nearing the end of life [8]. Small observational studies of PC within heart transplantation have demonstrated feasibility and a trend toward improved quality of life [9]. Studies have focused predominantly on how PC alleviates the emotional, physical, and spiritual distress that accompanies SOT [10,11]. Emerging literature also demonstrates notable mental health morbidity among those awaiting heart transplantation, with significant increases in the incidences of anxiety, depression, panic, adjustment, alcohol use, and eating disorders [12]. In particular, depression and anxiety appear to increase the risk for post-transplant mortality [13]. This case report discusses the management of a patient undergoing orthotopic heart transplant (OHT) evaluation with an initial preference for comfort-focused care, with PC involvement resulting in the clarification of goals of care and appropriate management of barriers, leading to an eventual successful OHT, which was in line with the patient's ultimate goals.

## Case presentation

A 67-year-old male with a past medical history of heart failure with reduced ejection fraction of 18% (New York Heart Association functional class IV) due to ischemic cardiomyopathy, myocardial infarction six years prior, chronic kidney disease stage three, cerebral vascular accident one year prior, and claustrophobia, initially presented to a community hospital with acute decompensated heart failure. He was transferred to a quaternary medical center for evaluation for advanced heart failure therapies, including left ventricular assist device and OHT, and initiated on continuous intravenous milrinone for support of his cardiac output. Per the transplant selection committee meeting, the patient was deemed to be a candidate for OHT pending completion of additional workup. However, upon further discussion with the cardiology team, the patient shared that the ongoing intensive care unit (ICU) level of care required to continue the transplant process was not aligned with an acceptable quality of life for him, and he requested to be discharged home as quickly as possible. PC was consulted to assist in furthering goals of care conversations. 

Upon initial assessment, the patient demonstrated a general understanding of the severity of his heart disease. However, he incorrectly believed that his prognosis would not be shortened by his desire to discontinue life-prolonging therapies, such as the continuous milrinone infusion, and reiterated his request to go home without completing OHT evaluation. Upon further exploration, he reported a long history of debilitating claustrophobia stemming from being trapped in an elevator in his 30s. His claustrophobia was significantly exacerbated in the hospital as he felt restrained by medical equipment such as intravenous lines, blood pressure cuffs, and his patient identification wristband. He denied seeking psychiatric care prior to this admission as he had previously been able to avoid triggers. During the OHT evaluation, the transplant psychiatry team initially prescribed alprazolam as needed for his claustrophobia, which the patient had only been taking intermittently due to his preference to minimize medication use. 

The PC team provided comprehensive guidance regarding what de-escalation of ICU level care would entail and clarified that he had a shorter prognosis of weeks to months without continuing life-prolonging therapies such as the milrinone infusion. In line with his initial request, the PC team provided anticipatory guidance and education regarding comfort-focused care and hospice services. When the patient was asked whether his priorities would shift if his claustrophobia could be better managed, he responded, "I came to the hospital for a reason; I want to live." His family affirmed that his goals were aligned with life prolongation but had been significantly hindered by his severe claustrophobia. The PC team further explored his uncontrolled symptoms, provided emotional support, and offered treatment recommendations to work toward achieving his goal of pursuing life prolongation through OHT. The patient agreed to proceed with a multi-modal approach including psychiatry, psychology, and integrative therapy team evaluations. 

The psychiatry team re-evaluated the patient and started scheduled trazodone and diazepam nightly in addition to gabapentin as needed. The psychology service met with the patient and completed 9 out of 12 attempted sessions, with the goal of visiting the patient twice a week during the acute peri-transplant phase. Treatment approaches included motivational interviewing, acceptance and commitment therapy, dialectical behavior therapy, and supportive therapy. Desired aims of therapy were to reduce anxiety, improve family relationship dynamics, and make claustrophobia a manageable condition. Conventional exposure therapy was not feasible due to the complex ICU setting. The patient was able to identify his strengths and practice relaxation techniques. He became invested in managing his claustrophobia, knowing that the chance to achieve OHT depended on it. He tolerated placement of an Impella catheter-based ventricular assist device and a prolonged ICU stay with fluctuating pain and other symptoms, while processing and reframing his fears and feelings of confinement. 

The patient successfully underwent OHT 42 days after initial PC evaluation. He was discharged home on postoperative day 10. In a subsequent transplant clinic visit three months later, the patient endorsed ongoing, though tolerable, claustrophobia. 

## Discussion

Patients undergoing OHT frequently experience psychological distress as they navigate a course that carries a high risk for morbidity and mortality. In the pre- and early post-transplant period, psychological issues can arise in relation to loss of independence and previous role identity, demoralization, adjustment to medical interventions, side effects of treatment, and concerns about surviving transplantation [14]. Furthermore, in the population awaiting heart transplant, 37% suffer from active psychopathology, with approximately half of these patients receiving an incidental psychiatric diagnosis during evaluation for OHT [15]. Uncontrolled psychopathology is a long-established driver of adverse outcomes in heart transplant patients [16]; thus, evaluation for OHT includes assessments of psychological readiness for transplant to mitigate these adverse outcomes [17]. In addition to impacting patient experience and transplant outcomes, psychological distress and psychopathology can interfere with goal-concordant care, as seen in this clinical case. It is not uncommon for patients with serious illness to voice a desire to defer life-prolonging treatments when in extreme distress or faced with uncontrolled symptoms, both physical and non-physical [18].

**Figure 1:**
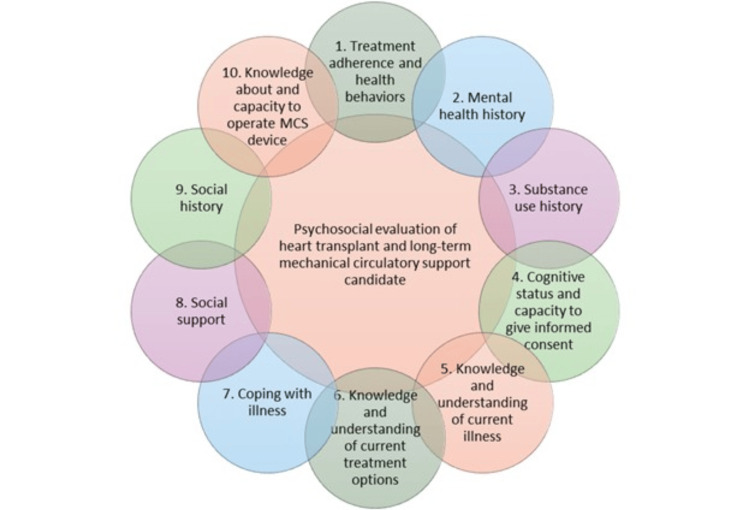
Consensus recommendations for domains to be assessed in the psychosocial evaluation of heart transplant and long-term mechanical circulatory support candidates. MCS: mechanical circulatory support Adapted from [19]

A key component of the initial transplant evaluation is a psychosocial evaluation, which includes 10 domains as described by recent consensus-based recommendations to promote consistency across clinical programs (Figure 1) [19]. It is recommended that evaluators consider utilizing templates or checklists when completing a psychosocial evaluation in order to systematically collect and report information. It is important for each transplant program to develop its own detailed protocol to address program-specific criteria and considerations [19]. In this clinical case, the PC team utilized serious illness communication techniques to further explore the patient’s distress, values, and goals [20]. As summarized in Table 1, these techniques can also be utilized by primary evaluators or treatment teams during the initial psychosocial evaluation or when caring for patients in times of acute distress.

**Table 1:** Serious illness communication techniques. Note: Adapted from [20]

Palliative care intervention	Rationale
1. Ask permission to start the encounter either as part of the transplant evaluation process (if built into the initial encounter) or by acknowledging the challenging time the patient is experiencing. It is recommended to meet in a private and quiet space and include family/friends for support, if allowed by the patient.	This encounter often sets the tone for the entire relationship that ensues. Ensure the physical environment respects patient privacy. Anticipate that the patient/family may become emotional during the conversation.
2. Provide empathetic listening and explore the patient’s experience and distress – one example is the NURSE mnemonic (Name, Understand, Respect, Support, Explore) [20].	This communication technique helps treatment teams to identify emotional distress and build rapport. In addition, it helps the patient feel supported and validated.
3. Allow for moments of silence, especially right after delivering a significant medical update or difficult news.	An invited emotional space for patients to process their emotions minimizes the psychological isolation that patients experience when they hear new information. Resist the urge to tell the patient how to feel. Give the patient time to absorb the information and respond.
4. Understand the patient’s unique personal values and background – this includes life outside of the hospital, support system, occupation, hobbies, spirituality, hopes, and worries.	Understanding patient values and hopes is valuable as a foundation for treatment teams to develop a goal-concordant care plan and to make patient-centered treatment recommendations. It is also helpful to identify personal strengths that will help patients to navigate anticipated difficult times, along with maladaptive coping mechanisms for which patients will need additional support.
5. Assess and inquire about the patient’s understanding of their disease process, current hospitalization (if pertinent), and proposed treatment options.	This information helps treatment teams to determine the patient’s perception of the medical situation and correct any misconceptions or misunderstandings. It is important to note if incongruent understanding is related to denial, emotional distress, or other coping mechanisms.
6. Refer to different medical subspecialists and multidisciplinary team members, as indicated.	Subspecialists provide further clinical assessment, support, and resources, and can include: psychiatry and psychology teams to treat emotional and psychiatric distress; palliative care team to manage refractory symptoms, provide psychosocial support, establish goal-concordant care plans, and navigate treatment teams’ ethical and moral distress; social workers, case management, spiritual care, integrative therapy, pet therapy, and volunteers to provide additional patient-specific support and interventions.

There is an increasing recognition that PC involvement is crucial in addressing key aspects of a patient’s care throughout the course of advanced heart failure and particularly during evaluation for OHT or in the cardiac critical care setting. With the ability to navigate both the psychosocial and medical realms, and an emphasis on understanding and supporting patient treatment preferences, PC can be an effective bridge between the many specialty and interdisciplinary teams involved in the transplant evaluation process. In this particular case, the PC team augmented the initial psychosocial evaluation and clarified the patient’s goal to successfully undergo OHT. The patient was referred to appropriate psychologic and psychiatric experts to support him in managing uncontrolled claustrophobia with treatment strategies such as long-term supportive psychotherapy and dialectical behavior therapy [19]. 

A recent statement from the AHA suggests that the optimal model of caring for patients undergoing transplant evaluation or in the cardiac critical care setting is a hybrid of primary and secondary (specialty) PC [13]. Primary PC is provided by the primary medical team and includes basic pain and symptom management, exploration of patient goals and values, facilitation of early prognosis discussions, and recommendation of limits to life-sustaining therapies if appropriate. As previously discussed, Table 1 includes serious illness communication techniques that primary teams can utilize in their own primary PC interventions. Indications for referral to specialty PC teams include the need for refractory symptom management, challenging psychosocial or family dynamics, complex goals of care discussions, and navigation of complex caregiver or treatment team moral distress [13].

## Conclusions

Patients with advanced heart failure, particularly those undergoing heart transplant evaluation, often experience psychosocial stress and exacerbation of psychopathology. It is best to consider the psychosocial evaluation as a process and not a single isolated event - enriched by multidisciplinary support to navigate patient and health-related issues - to improve quality of life, clinical outcomes, and tolerance of the complex process of heart transplantation. PC can function as a bridge between psychosocial and medical teams, with expertise in communication facilitation and symptom management, to ensure that care being delivered for this high-risk population remains patient-centered and goal-concordant. Based on this patient case, it may be helpful to study the feasibility of automatic PC involvement early on in the OHT evaluation process and its impact on patient outcomes and quality of care, with a focus on alleviating mental health morbidity.
